# Multibit memory operation of metal-oxide bi-layer memristors

**DOI:** 10.1038/s41598-017-17785-1

**Published:** 2017-12-13

**Authors:** Spyros Stathopoulos, Ali Khiat, Maria Trapatseli, Simone Cortese, Alexantrou Serb, Ilia Valov, Themis Prodromakis

**Affiliations:** 1 Department of Electronics and Computer Science, Faculty of Physical Science and Engineering, University of Southampton, University Road, SO17 1BJ Southampton, United Kingdom; 2 Forschungszentrum Jülich, Wilhelm-Johnen-Straße, 52428 Jülich, Germany

## Abstract

Emerging nanoionic memristive devices are considered as the memory technology of the future and have been winning a great deal of attention due to their ability to perform fast and at the expense of low-power and -space requirements. Their full potential is envisioned that can be fulfilled through their capacity to store multiple memory states per cell, which however has been constrained so far by issues affecting the long-term stability of independent states. Here, we introduce and evaluate a multitude of metal-oxide bi-layers and demonstrate the benefits from increased memory stability via multibit memory operation. We propose a programming methodology that allows for operating metal-oxide memristive devices as multibit memory elements with highly packed yet clearly discernible memory states. These states were found to correlate with the transport properties of the introduced barrier layers. We are demonstrating memory cells with up to 6.5 bits of information storage as well as excellent retention and power consumption performance. This paves the way for neuromorphic and non-volatile memory applications.

## Introduction

Resistive memory devices, also known as memristors^1^, are nowadays attracting considerable attention due to the breadth of potential applications ranging from non-volatile memory^2^ to neuromorphic systems^3,4^ and reconfigurable circuits^5^. Their competitive advantage over established complementary metal-oxide-semiconductor (CMOS)-based memory stems from their capability to support a multitude of states, long retention characteristics, fast switching and ultra-low power consumption^6^. Many technologies have been put forward as potential winners of the non-volatile memory race^7^, namely phase-change (PCRAM)^8^, magnetic (MRAM)^9^ and resistive random access memory (ReRAM)^10^. Although MRAM and PCRAM are considered more reliable, they are constraint by power and/or scalability issues^11,12^. In contrast, ReRAM has shown capacity of operating in the femtojoule regime^13^, with functional devices reported at feature sizes that outperform CMOS^14,15^. However, even though the realisation of bistable memory devices (1-bit) is apparent from the very nature of the memristor to variate between two resistive states^16^ the implementation of a device that can reliably be programmed at a multitude of distinct resistive states still poses a significant challenge. Although there are some recent reports of multibit capable metal-oxide memory cells^17^, most works in literature are limited to no more than 3 bits^18–22^.

Resistive switching has been observed in many metal-oxide systems^23^, with Ta_2_O_5_^24,25^, HfO_2_^26^ and TiO_2_^27,28^ being among the most popular. In all cases, the origin of switching has been attributed to either the drift of oxygen vacancies^28^ and/or interstitials^29^ or the formation of conductive filaments^30^ within an active metal-oxide core under the influence of an applied electrical field. Within that context several studies have reported on the importance of interface interactions and properties^31^, showing that the introduction of a thin interfacial barrier layer between the active layer and one of the electrodes can influence the electrochemical processes, the devices’ stability^32,33^, improve its switching characteristics and reduce the overall power consumption^34–37^.

**Figure 1:**
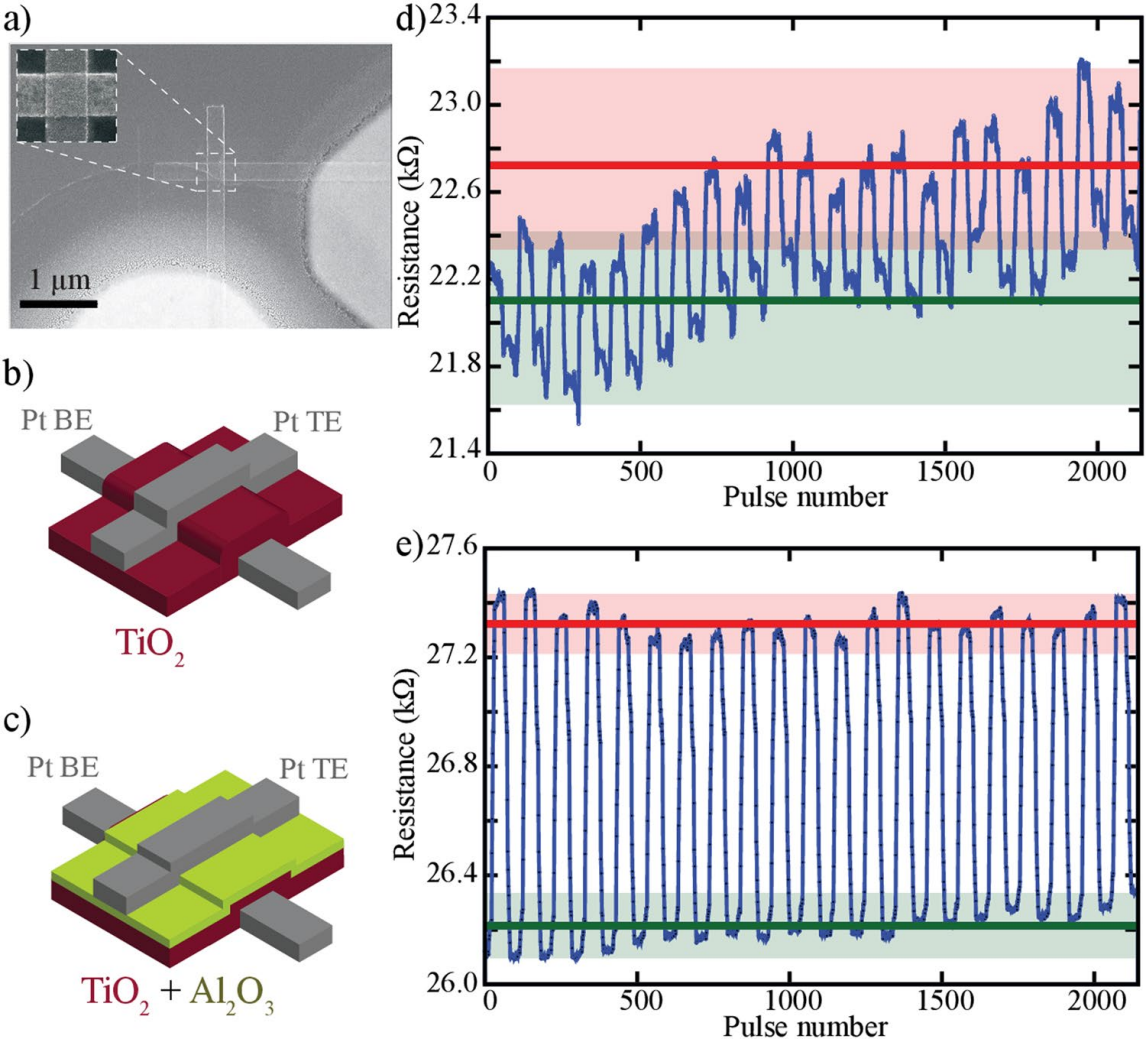
Comparison between TiO_2_-only devices and Al_x_O_y_/TiO_2_ bilayer devices. (**a**) SEM micrograph of a memristor device; (**b**) Schematic representation of a single layer TiO_2_-based device with platinum top and bottom electrodes; (**c**) Schematic representation of a bilayer Al_x_O_y_/TiO_2_-based device with platinum top and bottom electrodes; (**d**) Typical bipolar switching of a device based on the stack pictured in (**b**) using 100 ns pulses of alternating polarity voltage ramps ranging from 1 to 2 V, with voltage steps of 200 mV; (**e**) Typical bipolar switching of a device based on the stack pictured in (**c**) using 100 ns pulses of alternating polarity voltage ramps ranging from 1 to 2 V with voltage steps of 200 mV. The coloured horizontal lines in fig. (**d**) and (**e**) denote the average low (LRS) and high resistive state (HRS).

Taking into advantage these observations, we developed a series of 2-terminal prototype metal-insulator-metal (MIM) ReRAM cells, as depicted in Fig. 1a–c, with bilayer structure using TiO_2_ as solid electrolyte and seven different interface barrier layer configurations; all employing Pt top and bottom electrodes. The active layers studied were: 1) TiO_2_-only, 2) Al_x_O_y_/TiO_2_, 3) Ta_x_O_y_/TiO_2_, 4) SiO_2_/TiO_2_, 5) ZnO/TiO_2_, 6) HfO_x_/TiO_2_ and 7) WO_x_/TiO_2_. For all fabricated devices the thickness was maintained to 4 nm and 40 nm for the barrier and TiO_2_ layer respectively. The size of the devices used in this paper is 20 × 20 μm^2^. The TiO_2_ layer is amorphous and stoichiometric as more details can be found in our previous^38,39^. Smaller and larger area devices, namely 10 × 10 μm^2^ and 20 × 20 μm^2^, were considered but no apparent impact on the multibit performance was observed. All prototypes were electroformed with 1 μs pulses of negative polarity ranging from −3 to −12 V in steps of 100 mV, to an operational resistive state range, typically between 20–150 kΩ, depending on the stack configuration (see Supplementary Fig. S1). Figure 1d,e illustrate the difference in resistive programming stability between the TiO_2_-only and Al_x_O_y_/TiO_2_ ReRAM cells. In both cases, 100 ns pulse ramps of alternating polarity from 1 to 2 V with 200 mV step are used as input stimulus. Considerable drift in programming can be observed in Fig. 1d for the TiO_2_-only devices, which practically results into non-discernible memory states even after 20 switching cycles. Although the stability of TiO_2_-only devices can be further optimised at the expense of programing energy (see Supplementary Fig. S2), the comparable Al_x_O_y_/TiO_2_ cells indicate a more stable behaviour, as observed in Fig. 1e, overall maintaining a constant OFF/ON resistive ratio throughout the experiment. The more clear definition of low and high resistive states is similar to what Yu *et al*. have reported for the HfO_x_/AlO_x_ system^34^ in comparison to the respective single layer cell.

**Figure 2:**
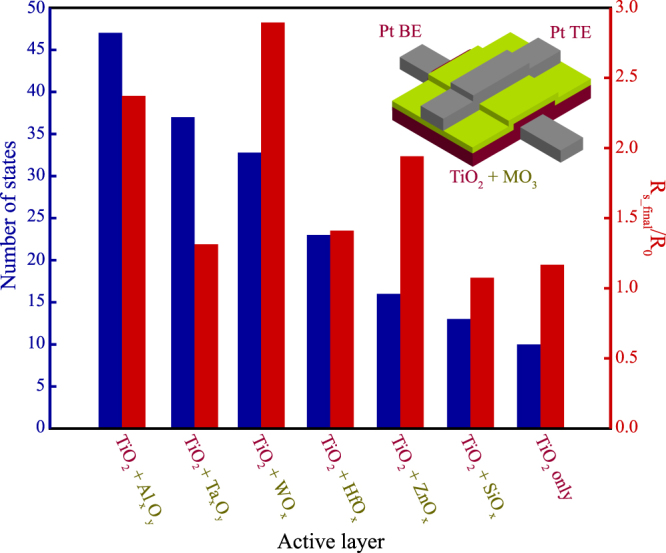
Multibit evaluation of devices based on different barrier layer combinations. Number of attainable resistive states (left axis) and ratio of the final state resistance over the baseline resistance (right axis) for typical bilayer devices. Confidence interval for the state assessment is 2σ following the routine described in Supplementary Figs S4 and S5. A chart containing each individual state assessed for every bilayer combination can be found in Supplementary Figs S6 and S7.

The need for stable and reliable switching at minute resistive increments becomes increasingly important if one wishes to exploit such cells as truly analogue memories. To that end, we evaluated all prototyped devices for their multistate capacity via biasing them with 100 ns pulses ranging from 1 to 2 V, at 50 mV steps. In every programming cycle, a new state is assumed to have been reached if two conditions are met. First, the resistive state is sufficiently stable over time, as evaluated by retention testing. Second, the lower bound of the standard deviation of a series of 50 × 0.5 V read pulses is at least 2σ higher than the upper bound of the previous state (see Methods and Supplementary Figs S3–S5 for more detail). Using this evaluation routine, we observed a significant increase in the number of attainable resistive states for the bilayer devices in contrast to the single-layer cells. While in the case of TiO_2_-only devices a maximum of 10 states on average was identified, the introduction of a barrier layer resulted into both increasing the number of resistive states significantly but also improving the dynamic response of the devices. Figure 2 summarises the switching performance of all developed bi-layer ReRAM cells both in terms of the number of attainable memory states and the resistive state dynamic range. All device prototypes that encompass an active bi-layer show improvements in both performance metrics.

**Figure 3:**
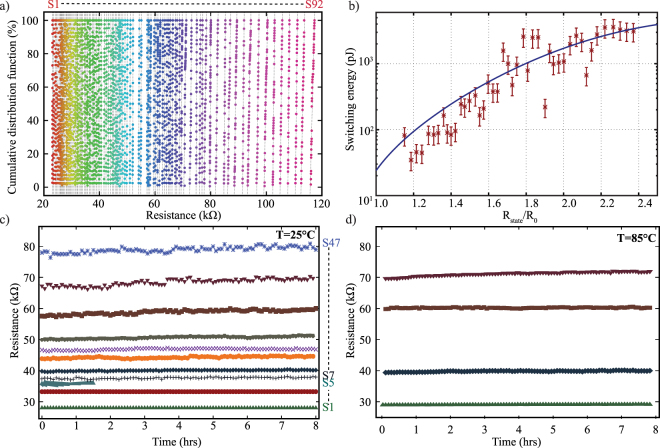
Multibit operation of a device using the Al_x_O_y_/TiO_2_ RRAM stack. (**a**) Cumulative probability distribution function plot of a device with a record of 92 distinct resistive states. All states are read at 0.5 V, are closely packed and individually discernible; (**b**) switching energy required to switch a typical Al_x_O_y_/TiO_2_ device. Only the energy expended during programming is regarded for this graph; (**c**) 8 hours retention measurements for select resistive states at room temperature. (**d**) 8 hours retention measurements for select resistive states at 85 °C. Resistance can be retained even at elevated temperatures.

The performance of the Al_x_O_y_/TiO_2_ devices is exemplified in Fig. 3a where a record number of 92 states is reported, which corresponds to a single cell with 6.5 bits memory capacity. This cumulative probability distribution function graph clearly illustrates the overall discernibility of the resistive states. Retention characteristics of a selection of states of a typical Al_x_O_y_/TiO_2_ device are shown in Fig. 3c,d over a period of 8 hours at room temperature and at 85 °C. It can be observed that the stored memory states are stable and remain clearly distinguishable even in the 30–40 kΩ range where the states are closely packed.

**Figure 4:**
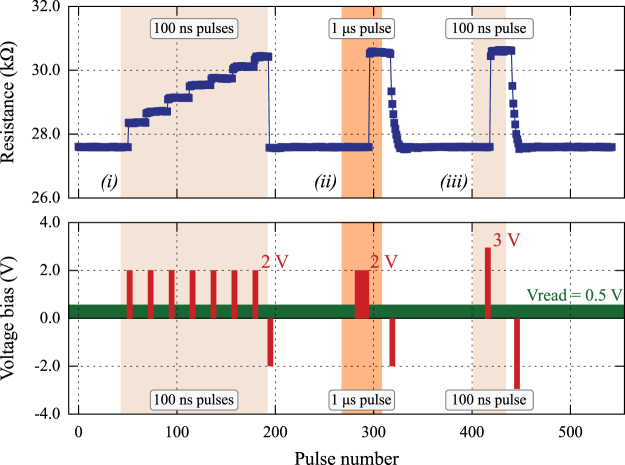
Programming the Al_x_O_y_/TiO_2_ device. Modes of selecting specific resistive states. Starting from a baseline resistance of ~27.5 kΩ a specific resistive state can be attained with different modes of programming, by modulating (i) the number of pulses, (ii) the duration of the pulses or (iii) the amplitude of the pulse. Using multiple pulses of lower amplitude and pulse width can help in pinpointing resistive states that otherwise could not have been discerned. In between the state selection the device is flushed with a series of 100 RESET pulses of 100 ns in duration.

The Al_x_O_y_/TiO_2_ combination proved to yield the best analogue performance given the “state expanse” figure of merit: (max{*R*/*R*_o_} × (# of states), where *R*_*o*_ is the baseline resistance. The improved stability allows us the programming of such elements in an arbitrary manner, as shown in Fig. 4. More specifically, single 100 ns wide pulses at 2 V allow us to sequentially set the resistive state of the device gradually. Selection of a different memory state can be done by first “flushing” the device back to its baseline resistance (27.5 kΩ) via a train of 100 ns wide RESET pulses at −2 V and then applying a corresponding number of SET pulses to reach the desired memory state. The resistive state of the device can also be selected by modulating not only the number of pulses but the duration or the amplitude of the programming pulse. As Fig. 4 shows by modifying the duration of the pulse or the amplitude similar high resistive state to the sequential pulsing can be exhibited clearly illustrating the time-voltage dilemma. However the resolution of the device suffers as several resistance levels are suppressed. It is apparent that using smaller, more incremental and precise pulsing steps makes extracting more usable resistive states from the memory cell possible.

The remarkable analogue memory performance and stability of states can be attributed to the specific ratio of the ionic transference numbers of the second oxide layer. By observation of the data shown in Fig. 2, a clear trend can be identified for the number of available states, whereas no particular trend on particular dependence can be observed. The highest number of stable non-volatile resistive states is achieved with the introduction of Al_2_O_3_, followed by Ta_2_O_5_, WO_3_, HfO_2_, ZnO and SiO_2_. It has been recently shown that many oxide thin films used for ReRAMs have mobile host cations^29^ and that as expected the oxidation state and stoichiometry of the matrix is also playing a significant role^40^. Mobility of cations and anions during high field oxide formation on metals using liquid electrolytes is well known from classical electrochemistry. In high voltages and low film thickness conditions, the transport is field-accelerated and the particular ionic transference numbers depend on the field. Al_2_O_3_ is identified as having the highest cation transference number, followed by Ta_2_O_5_, WO_3_ and HfO_2_^41–43^. The identified trend in the data of Fig. 2 strictly correlates with the higher mobility of cations or lower mobility of oxygen ions, respectively. Similar effect of the oxygen mobility on the device stability has been reported for STO using barrier layers of Al_2_O_3_ (low O^2−^ mobility) and yttria-stabilized ZrO_2_ (high O^2−^ mobility)^44^. We can therefore conclude that the main factor influencing the observed device performance is attributed to the transport properties of the interfacial film added to the TiO_2_ layer.

It is important to mention that our characterisation routine foregoes the use of compliance current limiting, while toggling between resistive states. Current compliance limiting is a common practice that is used to control the size of the conductive filament and consequently the overall resistance of the device^45,46^. Instead, we have opted for a more direct approach by sequentially pulsing the device until its state stabilises. As the energy budget is increased incrementally until the resistance exceeds a predefined tolerance, we ensure that the minimum amount of required switching energy is expended. Figure 3b depicts the calculated programming energy requirements of a typical Al_x_O_y_/TiO_2_ device with 47 distinct states. An upper bound of the energy consumption per state during programming can be estimated as ${\sum }^{}\{{V}^{2}/{R}_{min,max}{\rm{\Delta }}t\}$, where *V* is the programming pulse voltage amplitude and ∆t the pulse width. As biasing typically occurs between 1 and 2 V, *R*_min_,_max_ represent the resistance in these two voltages as calculated from the I–V characteristic in the low resistive state (see also Fig. S8). For all the states of the Al_x_O_y_/TiO_2_ device the switching energy remains in the pJ–nJ range. Even though the overall OFF/ON ratio of the devices among the resistive states is small in comparison to previous publications^47,48^ the incremental biasing steps used in this paper allow for further exploitation of the resistive values of the device that would otherwise not be possible with larger ratios. In addition our approach alleviates issues of poor retention performance mentioned in these works^47,48^.

**Figure 5:**
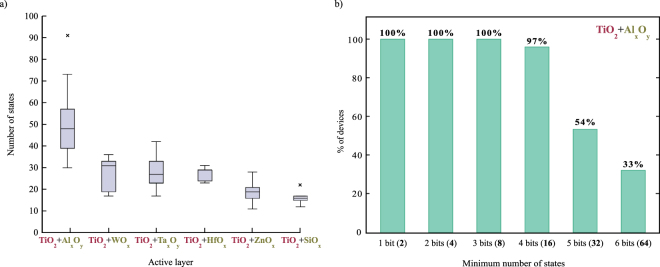
Bilayer device statistics. (**a**) Number of resistive states ranges for different bilayer combinations. Al_x_O_y_/TiO_2_ devices clearly outperform all other combinations with ZnO_x_/TiO_2_ and SiO_x_/TiO_2_ exhibiting the lower amount of resistive states; (**b**) Attainable states distribution for 32 Al_x_O_y_/TiO_2_ based devices. Nearly all the devices tested exhibit at least 4 bits of information whereas 1/3 of them surpasses the 6-bit mark.

As far as the statistical distribution of the resistive states for different bilayers is concerned we can observe in Fig. 5a that Al_x_O_y_/TiO_2_ bilayer clearly outperforms the other combinations with a median of 47 states (5.5 bits), followed by WO_x_/TiO_2_ and Ta_x_O_y_/TiO_2_ with a median in the 4-bit range and finally by ZnO_x_/TiO_2_ and SiO_x_/TiO_2_ in the 3-bit range. Even in the worst case the Al_x_O_y_/TiO_2_ devices consistently exhibit at least 4-bit of information with half of the devices surpassing 5-bits and 1/3 the 6-bit mark (Fig. 5b).

In this paper, we demonstrated that the incorporation of different metal-oxide barrier layers in ReRAM improves the overall programming stability. This is enabled via the improved transport properties of the device depending on the increasingly higher mobility of cations and subsequent lower mobility of oxygen ions, in accordance to the employed barrier layer. Through this study, we were able to demonstrate for the first-time solid-state ReRAM operating as analogue memory cells with up to 5.5-bits capacity. While ReRAM technologies have been mainly promoted for high-spatial density storage and corollary applications, our work demonstrates the new prospects arising from high-capacity memory.

## Methods

### Device fabrication

All devices have been fabricated on 6-inch oxidised silicon wafers (200 nm of thermal SiO_2_). Initially the bottom electrodes were fabricated using photolithography and electron beam evaporation of titanium (5 nm) and platinum (10 nm) followed by lift-off process in N-Methyl-2-pyrrolidone (NMP). Then, 40 nm of TiO_2_ were deposited using magnetron sputtering. The Al_2_O_3_, Ta_2_O_5_, and SiO_2_ layers (4 nm) were also deposited using magnetron sputtering after negative tone photolithography. The active layer is formed after lift-off in NMP. The 4 nm layers of ZnO, HfO_2_ and WO_3_ were synthesised using atomic layer deposition (ALD). After that a positive tone photolithography and ion beam milling processes were used to pattern and etch the active layers. The top electrode was fabricated using photolithography, electron beam evaporation of platinum (10 nm) and lift-off in NMP.

### Electrical characterisation

Characterisation of the memristors has been done with our in-house memristor characterisation platform^49^. All read pulses are set at 50 ms in duration and 0.5 V in amplitude. Nominal line resistance for all devices evaluated is estimated to be about 150 Ω per platinum electrode. Devices are initially electroformed to a usable resistance range (25 to 200 kΩ, depending on the stack) using consecutive 1 μs pulses of negative polarity ranging from −8 to −12 V in amplitude. A series resistor of 1 kΩ was used as a current-limiting mechanism for all devices. Resistance initially drops to the 10^6^ Ω range and then to a more stable 10^4^–10^5^ Ω range. Multi-bit capability of the devices has been evaluated with a custom algorithm (see following section). In order to extract the retention curve a sequence of 100 ns 2 V pulses is used to program the device to a specified resistance and then a read pulse is applied every 5 minutes for 8 hours. For temperature dependent retention measurements resistive level was selected after 85 °C have been stabilised in the probe station chuck.

### Resistive state evaluation algorithm

State assessment occurs over three phases. During the first phase a series of programming pulses of a predefined duration (100 ns), increasing amplitudes and alternating polarities is applied to the device under test and the resistive state of the device is evaluated between every pair of programming trains. This is to determine the polarity that induces a switch in the resistance of the device. After the switching polarity has been determined the second phase, using fixed amplitude, 100 ns pulses of the opposite polarity in respect to the one determined in the first phase, drives the resistance to a stable low value. Stability is assumed when the fitted slope is lower that a predefined threshold. The third phase applies an increasing number of 100 ns programming pulsing using the polarity determined from the first phase followed by two read trains separated by a 100 ms retention interval. If the lower bound of the standard deviation of the resistance measured between these trains is at least 2σ higher than the upper bound of the previous state a new resistive state is established. The algorithm terminates if the voltage limit is reached or if the trend of the resistive states become non-monotonic. The granularity on the standard deviation directly impacts the number of assessed states (see Supplementary Fig. S3). 2σ was used throughout the electrical characterisation as it provides a large enough confidence interval (at least 95%) while allowing the exploitation of a high amount of resistive states. A flowchart detailing the steps of the algorithm described here can be found in Supplementary Fig. S4.

### Data Availability

The data that support the findings of this study are available from the University of Southampton institutional repository at https://doi.org/10.5258/SOTON/D0329.

## Electronic supplementary material

Supplementary information for manuscript
