# Supplementary material for: Multibit memory operation of metal-oxide bi-layer memristors
Source: Sci Rep. 2017 Dec 13;7:17532. doi: 10.1038/s41598-017-17785-1 (PMC5727485; doi:10.1038/s41598-017-17785-1)
Supplement: Supplementary file 1 — Supplementary information for manuscript [file 41598_2017_17785_MOESM1_ESM.pdf]

Multibit memory operation of metal-oxide bi-layer memristors
Supplementary information

Spyros Stathopoulos[1], Ali Khiat[1], Maria Trapatseli[1], Simone Cortese[1], Alexandrou Serb[1], Ilia Valov[2] and Themis Prodromakis[1*]

[1]Department of Electronics and Computer Science, Faculty of Physical Science and Engineering, University of Southampton, University Road, SO17 1BJ, Southampton, United Kingdom.

[2]Forschungszentrum Jülich, Wilheml-Johnen-Straße, 52428, Jülich, Germany

[*]Corresponding Author: Themis Prodromakis (Email: t.prodromakis@soton.ac.uk)

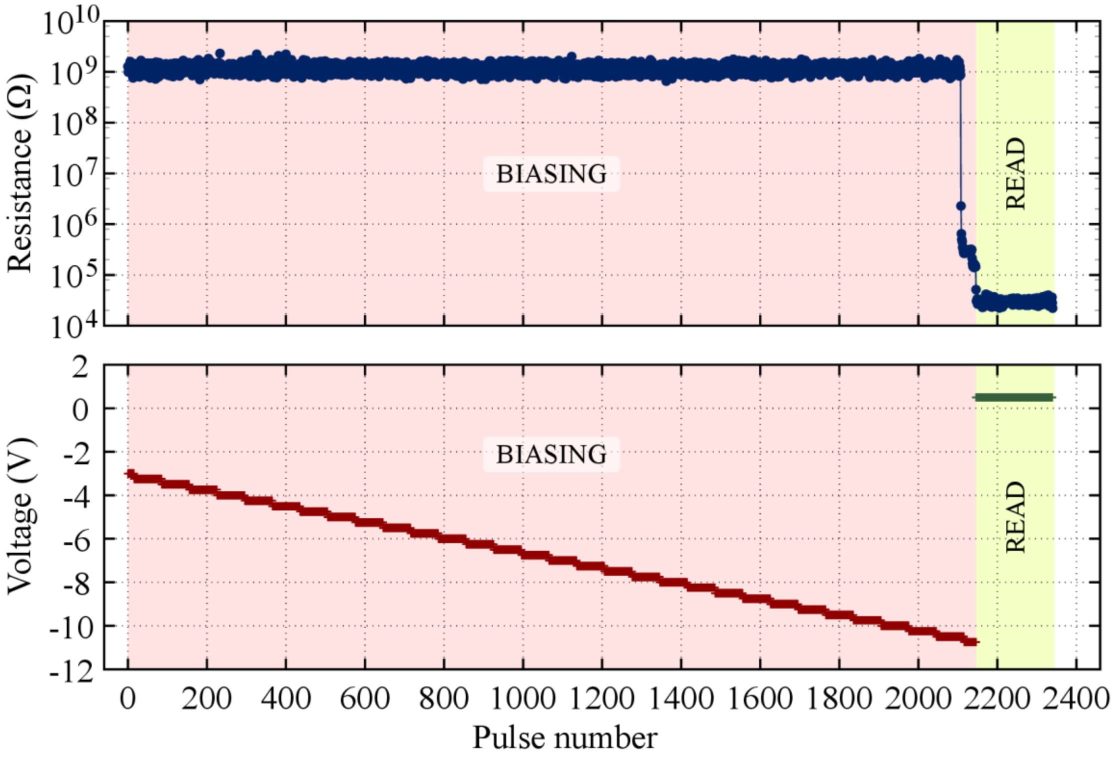

Supplementary Fig. S1: Electroforming behaviour of an Al_xO_y/TiO_2 device. Electroforming was performed using 1 μs pulses ranging from -3 to -12 V for all devices. A typical response to this electroforming protocol, exhibited here in an Al_xO_y/TiO_2 device, is an initial drop in the resistance (here at ~75 kΩ) at around 10 V followed by a further drop into the usable initial resistance range.

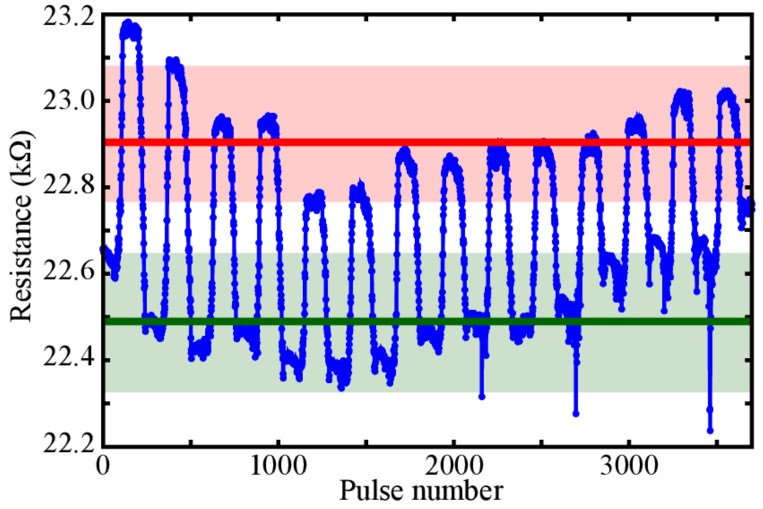

Supplementary Fig. S2: Optimised pulsing protocol for a TiO_2-only device. Stability of the TiO_2-only can be improved when using ramps of 1 μs voltage pulses ranging from 1 to 3 V with 100 mV step and alternating polarities. Although the required energy is increased (longer pulses, higher voltage) the stability of the TiO_2-based device improved to the point that the worst-case switching windows between low and high resistive states are non-overlapping.

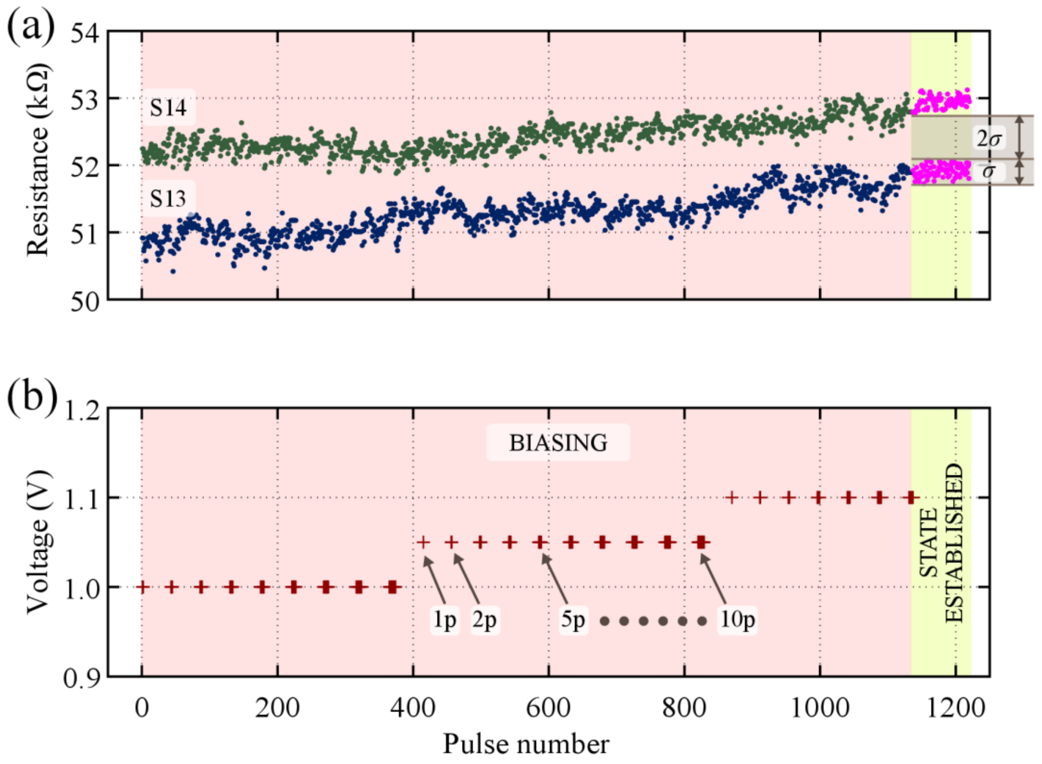

Supplementary Fig. S3: Assessment between two adjacent resistive states for a device using the Al_xO_y/TiO_2 stack. An increasing number (up to 10) of 100 ns programming pulses (b) is applied with 50 mV step. In-between the programming pulses there are 50×0.5 V read pulses. During the last 50 read pulses the lower bound of resistance of the device should be at least 2σ greater that the upper bound of the resistance of the previous state (51.86 ± 0.17 kΩ in the shown example) and therefore a new resistive state is established at 52.98 ± 0.14 kΩ. We observe a phase during which our test device

experiences a constant and very fine-grained resistance increase. This corresponds to the part of the testing routine designed to push the resistance -very gradually and controllably- to a value that can be readily accepted as distinct from the previous state and thus considered a truly new state.

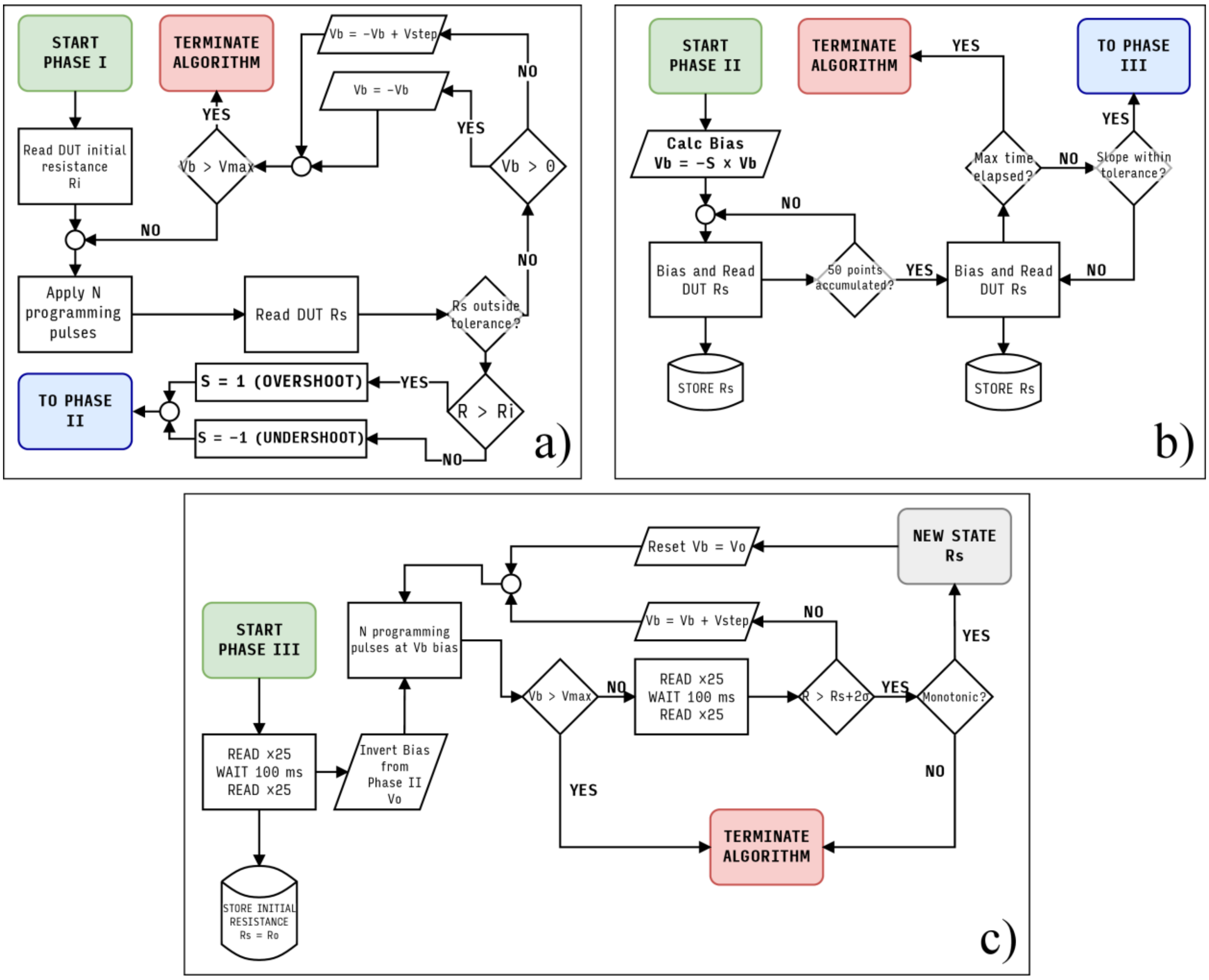

Supplementary Fig. S4: Block diagram of the state evaluation algorithm. State evaluation happens over three distinct phases. *(a) Phase I* determines the switching polarity of the device by applying pulses of alternating polarity. If the applied pulse causes a resistance response outside a predefined tolerance band the switching polarity *S* is determined to be either positive (if the final resistance is above the tolerance band) or negative (if the final resistance is below the tolerance band). *(b) Phase II* drives the resistance of the device to a stable low or high level. Depending on the outcome of Phase I a series of pulses of opposite polarity is applied until the slope of the fitted resistance response is less that a predefined threshold. A minimum of 50 points is accumulated for this evaluation. *(c) Phase III*: initially a base resistance and its standard deviation is calculated. This calculation is composed of two sets of 25 read pulses separated by a (configurable) retention time of 100 ms. Afterwards a train of pulses of constant voltage and width is applied using the polarity determined from Phase I. The resistance of the device is evaluated again using the same method as the one used for the base resistive state. A new state is established if the lower/upper bound of the standard deviation of the new resistive state is at least 2 or more standard deviations above the upper/lower bound of the standard deviation of the previous resistive state. Otherwise the voltage is increased and the process repeats. The algorithm terminates if a maximum voltage is reached or the resistive state sequence becomes non-monotonic

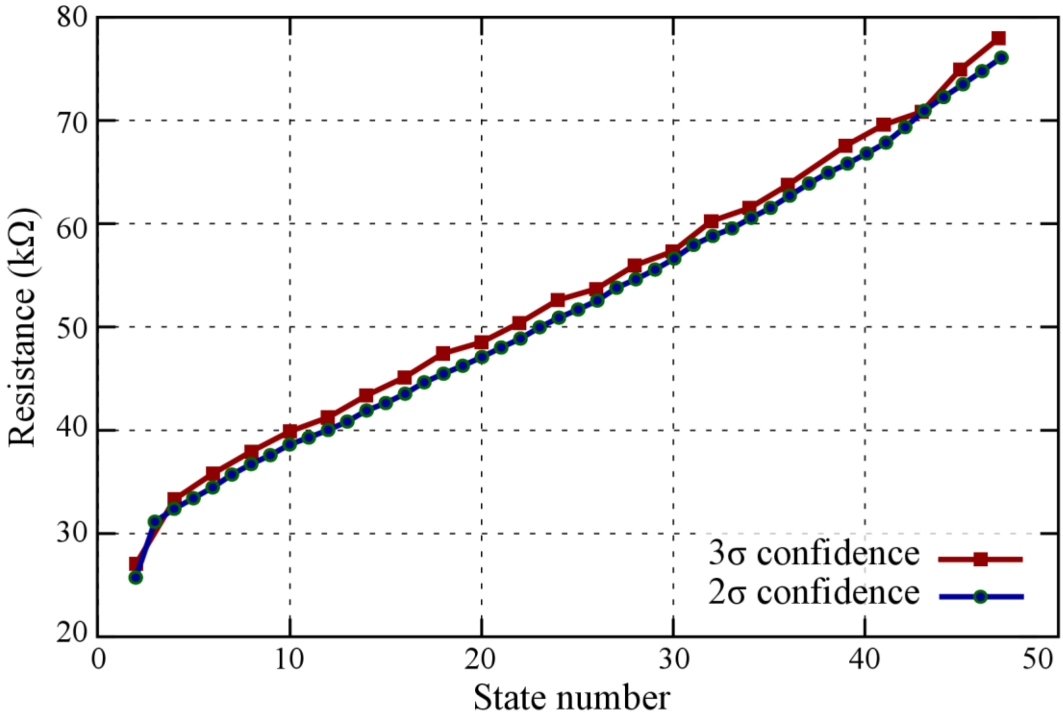

Supplementary Fig. S5: Effect of the confidence bounds in the number of attainable states of the Al_xO_y/TiO_2 device. The maximum number of possible attainable states depends on the confidence bounds used. By using 3σ (99.7%) instead of 2σ (95%) the number of registered resistive states is roughly halved (23 from 47). For most practical scenarios, however, a 95% confidence interval is sufficient to discern two adjacent states.

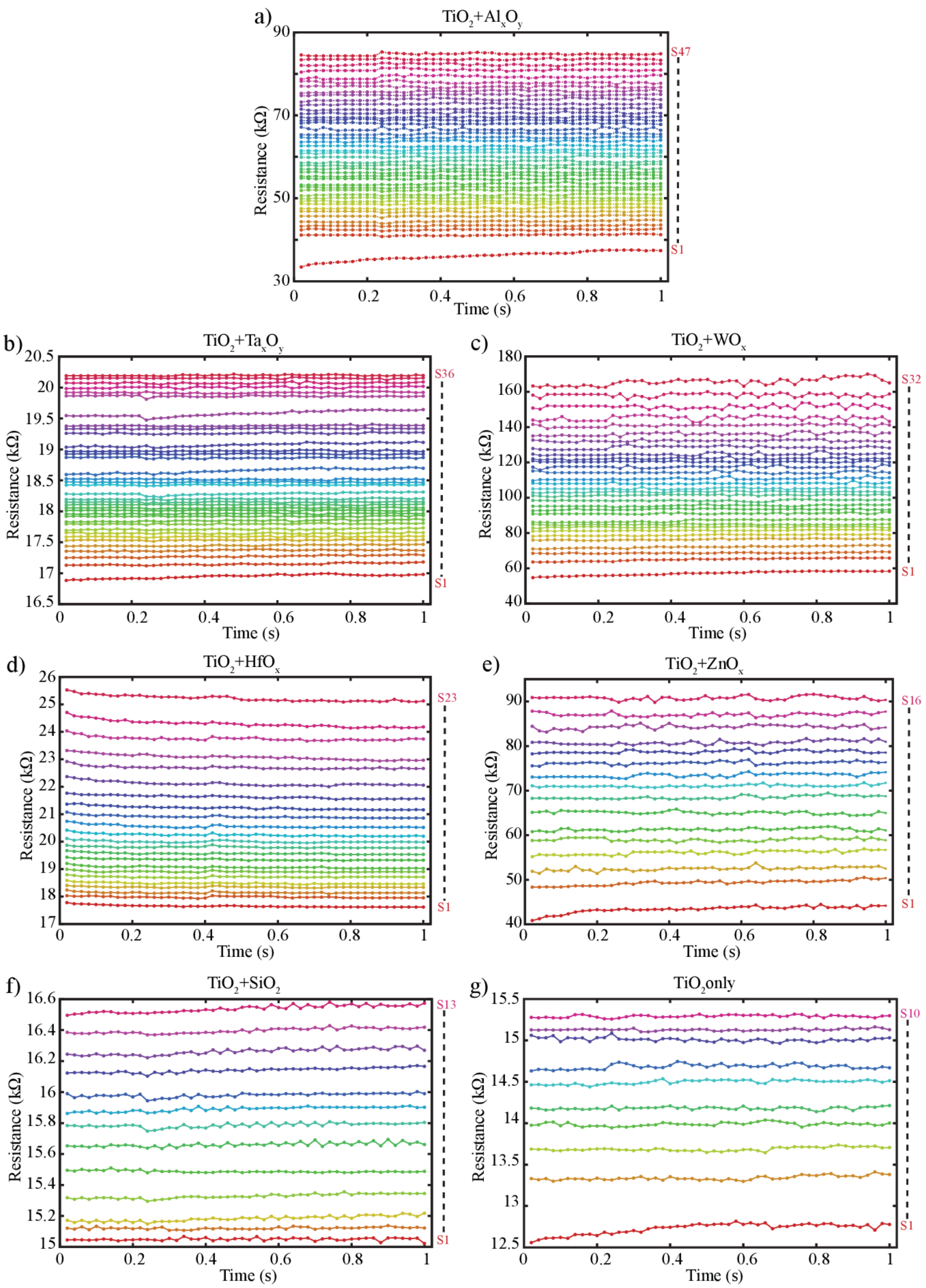

Supplementary Fig. S6: "Short term" retention for all bilayer combinations. The results of the final state measurements (50×100 ns pulses with 20 ms interval). With the exception of SiO_2 all bilayer combinations improve both the number of attainable states and the overall stability of each established state in comparison to the TiO_2-only device.

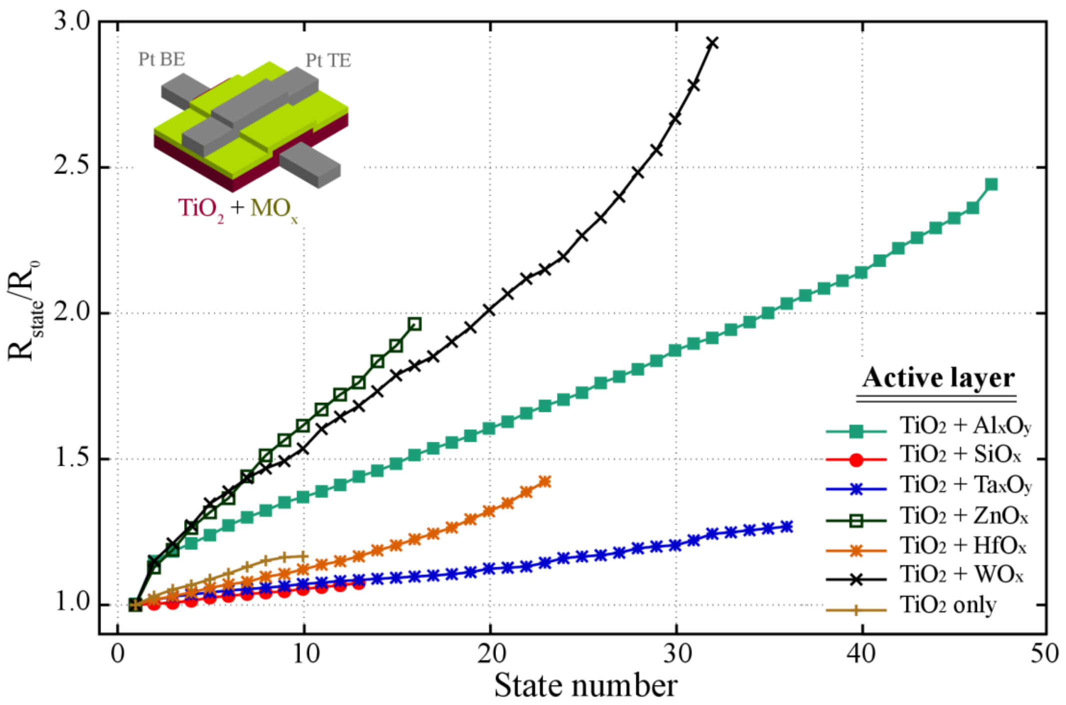

Supplementary Fig. S7: Multistate evaluation for different bi-layer combinations. The chart depicts all established resistive states with 2σ confidence for each bilayer combination studied in this paper. All combinations are improving the TiO_2 stack regarding the number of states but only Al_xO_y/TiO_2, WO_3/TiO_2 and HfO_2/TiO_2 stacks also provide an increase the dynamic range of the device. The 46 resistive states, the overall linearity as well as the improved dynamic range constitute the Al_xO_y/TiO_2 the most promising combination for granular, predictable, multi-bit storage.

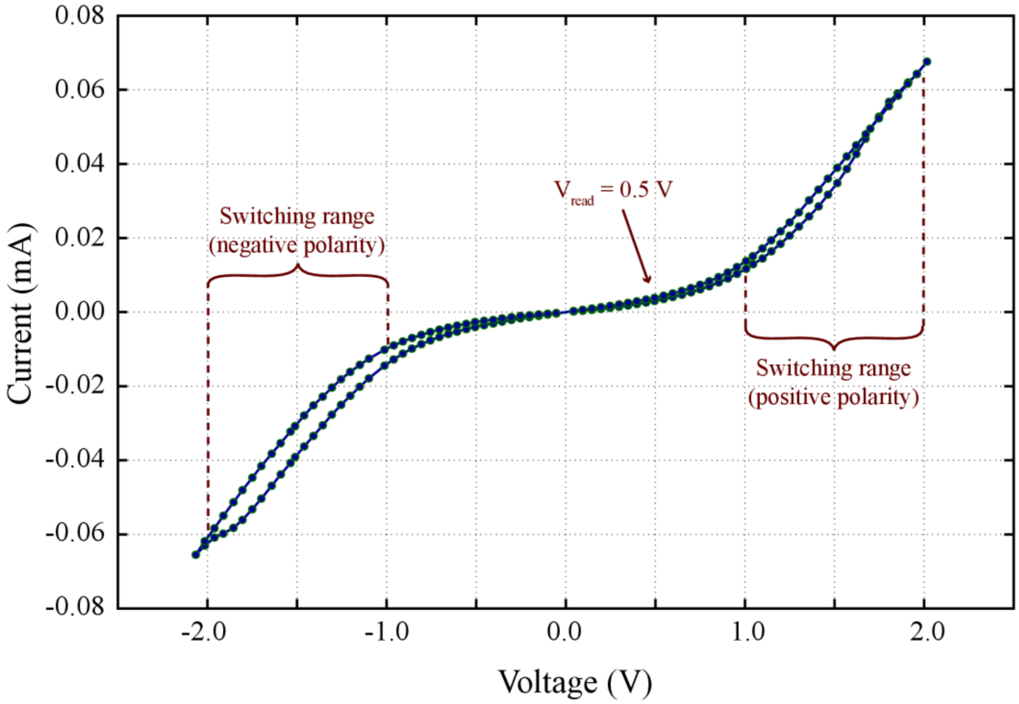

Supplementary Fig. S8: I–V characteristic of an Al_2O_3/TiO_2 device in the ~25 kΩ range. In order to calculate the required switching energy (see main text fig. 4b) the formula $\Sigma\{V^2/R_{min, max} \cdot \Delta t\}$ was used. R_{min} and R_{max} are extracted by multiplying the resistance at READ voltage (0.5 V) with the ratio of the slopes at 0.5 V versus 1 V (for Rmin) or 2 V (for Rmax). This is a very conservative "worst case" approach to estimate the energy usage for the device. Starting from the low resistive state, which is the most energy consuming state, we take a current–voltage characeteristic that covers the relevant switching range. Since we are starting from the base resistive state of the device the I–V shows no signs of further setting even when the voltage is up to 2 V therefore power dissipation for a device subjected to 2 V and setting must be necessarily lower that our assessment based on this I–V.

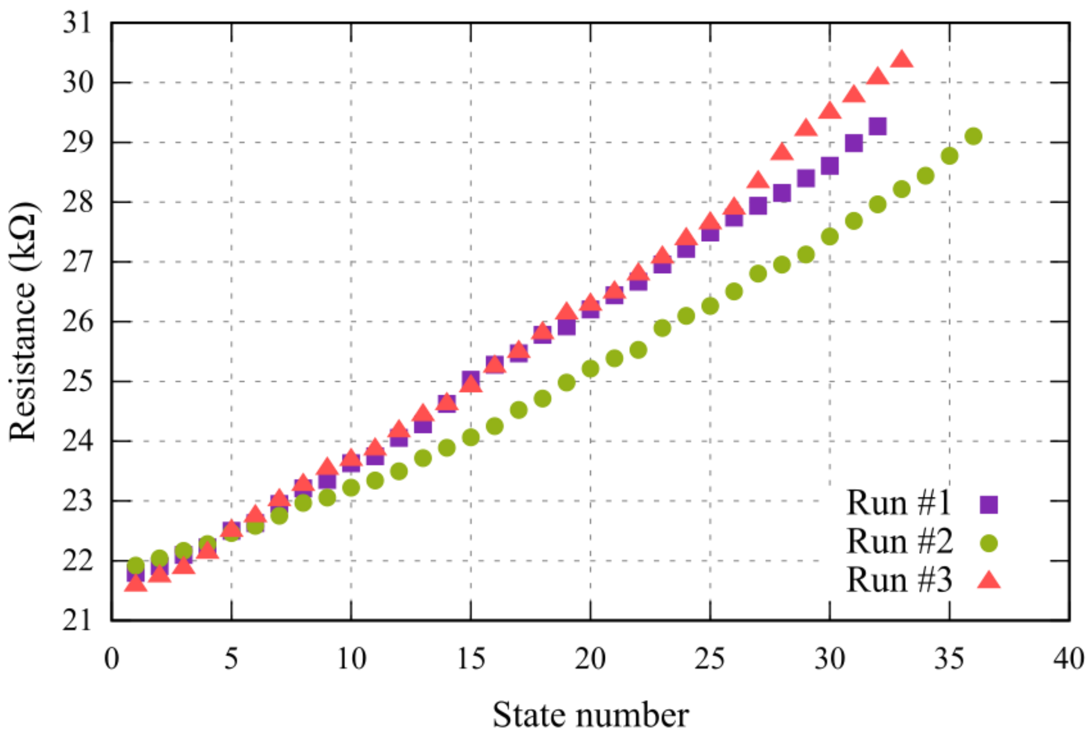

Supplementary Fig. S9: Consecutive multibit assessment routines. Three consecutive multibit characterisation routines are run to determine the repeatability of the number of states and the resistance range.

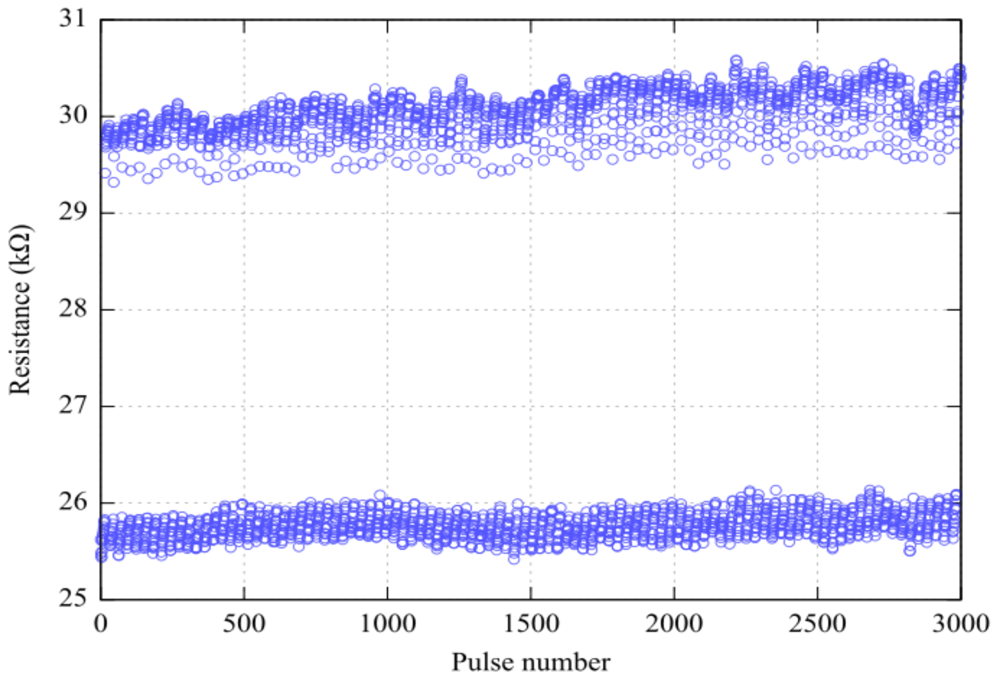

Supplementary Fig. S10: Endurance measurements. Endurance measurement for 3000 pulses using alternating sets of 15×1 μs pulses at 2 V.

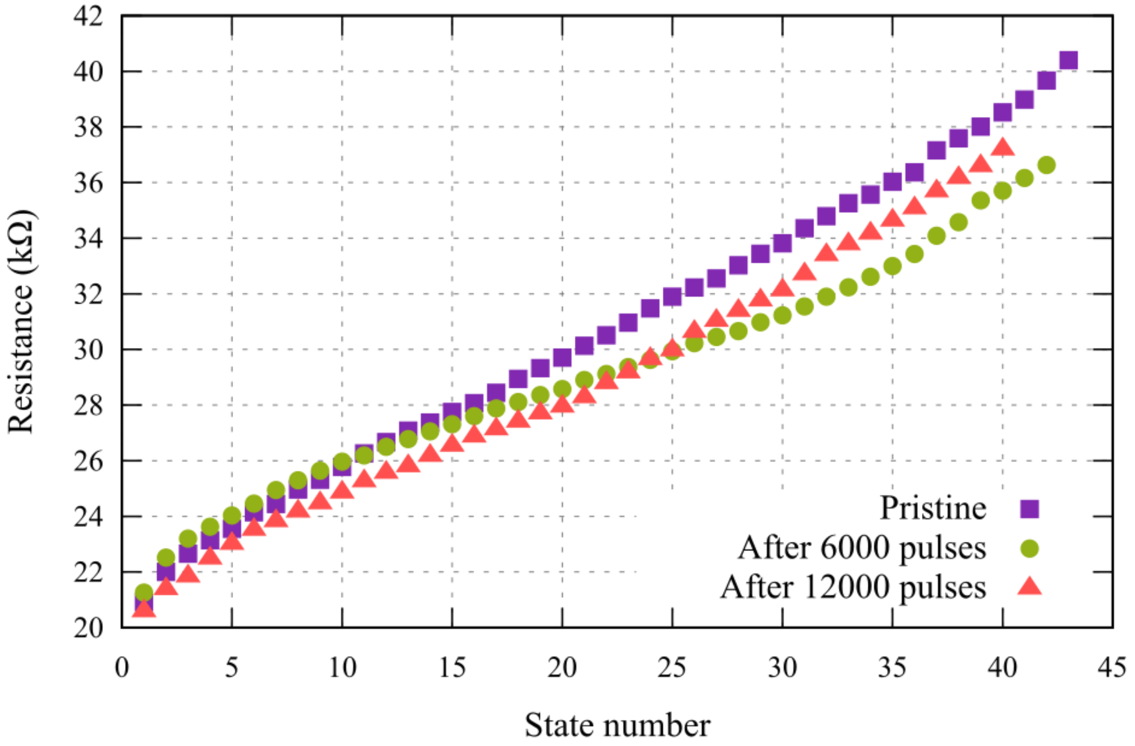

Supplementary Fig. S11: Number of attainable states after consecutive endurance cycles. Multibit evaluation of a memristive cell after cycling the device for 6000 and 12000 pulses using alternating sets of 15×1 µs pulses at 2 V.
